# The probability of having advanced medical interventions is associated with age in out-of-hospital life-threatening situations

**DOI:** 10.1186/s13049-016-0294-4

**Published:** 2016-08-24

**Authors:** Vania Tavares, Pierre-Nicolas Carron, Bertrand Yersin, Patrick Taffé, Bernard Burnand, Valérie Pittet

**Affiliations:** 1 Institute of Social and Preventive Medicine, Lausanne University Hospital, Lausanne, Switzerland; 2 Emergency Department, Lausanne University Hospital, CH-1011 Lausanne, Switzerland

**Keywords:** Ageing, Out-of-hospital emergency medical services, Cardiopulmonary resuscitation, Critical care medicine, Decision-making

## Abstract

**Background:**

The use of out-of-hospital emergency medical services by old and very old individuals is increasing. These patients frequently require complex evaluation and decision-making processes to determine a strategy of care, therapeutic choices or withdrawal of care in life-threatening situations. During out-of-hospital missions, thorough decision-making is difficult because of the limited amount of time and lack of direct access to medical charts or to pre-existing advance directives. In this setting, age may be used as a proxy to determine strategy of care, therapeutic choices or withdrawal of care, particularly in relation to advanced medical interventions. We aimed to determine how an emergency physician’s initiation of out-of-hospital advanced medical interventions varies with the patient’s age.

**Methods:**

We performed a retrospective analysis of the missions conducted by the emergency physicians-staffed emergency medical services in a Swiss region. We used logistic regression analysis to determine whether the probability of receiving an advanced medical intervention was associated with the patient’s age.

**Results:**

Among 21,922 out-of-hospital emergency adult missions requiring an emergency physician, the probability of receiving an advanced medical intervention decreased with age. It was highest among those aged 18 – 58 years and significantly lower among those aged ≥ 89 years (OR = 0.66; 95 % CI: 0.53 – 0.82). The probability of cardiopulmonary resuscitation attempts progressively decreased with age and was significantly lower for the three oldest age deciles (80 – 83, 84 – 88 and ≥ 89 years).

**Conclusion:**

The number of out-of-hospital advanced medical interventions significantly decreased for patients aged ≥ 89 years. It is unknown whether this lower rate of interventions was related only to age or to other medical characteristics of these patients, such as the number or severity of comorbidities. Thus, further studies are needed to confirm whether this observation corresponds to underuse of advanced medical interventions in very old patients.

## Background

The number of out-of-hospital emergency medical services (EMS) missions is increasing for people ≥ 65 years [1–5], reflecting population ageing and potential changes in patients’ and caregivers’ expectations, as well as structural changes in the health care system [5]. Older patients admitted to emergency departments (ED) are usually transported by ambulance and present with more serious situations than younger patients do, with a higher risk of mortality [1, 4, 6]. However, it is uncertain whether age is an independent factor influencing survival and functional status after specific acute conditions, such as out-of-hospital cardiac arrest. It has indeed been shown that age is a weak and inconstant predictor of survival rate [7, 8]. Moreover, studies have reported that the functional status of the survivors of an out-of-hospital cardiac arrest depended more on other factors, including comorbidities, a non-cardiac origin, or the presence of pre-existing dementia or functional dependency [7, 9]. Although most of these factors are associated with age, age did not appear to be an independent risk factor of poor prognosis [9]. Comorbidities, quality of life, and patients’ expectations were better predictors of outcome [10, 11].

During EMS missions, thorough decision-making is difficult because of the limited amount of time and the lack of direct access to medical charts or to pre-existing advance directives [12]. In this setting, age is still commonly used as a proxy to determine strategy of care, therapeutic choices or withdrawal of care [13]. In life-threatening conditions, dementia, age, and patients’ and relatives’ willingness for the patient to undergo advanced medical interventions (ADMI), along with the number of available hospital beds, are among the most important determinants in a clinician’s decision to admit a patient to the intensive care unit [14]. Other studies have pointed towards additional factors, such as functional dependence, an underlying incurable disease or limited life expectancy [14, 15].

In the present study, we evaluated whether the probability of receiving ADMI in a EP-staffed EMS varies with age in out-of-hospital emergency situations.

## Methods

### Study design and setting

This retrospective analysis was based on data routinely collected for each emergency physicians-staffed out-of-hospital EMS mission performed from 2005 to 2013 in the Canton of Vaud, Western Switzerland (~750,000 inhabitants at the end of 2013). In this region, a unique emergency dispatch centre coordinates the EMS. Staffed by trained nurses or paramedics, the centre uses a specific keyword-based dispatch protocol in which the patient’s age is not included. Trained paramedics constitute the initial response of the out-of-hospital EMS. Out-of-hospital emergency physicians (EP) may be dispatched in the case of life-threatening emergencies or at the request of the paramedics. EP missions may be cancelled by paramedics when they arrive on site first and face a non-emergency situation.

### Study population

**Fig. 1:**
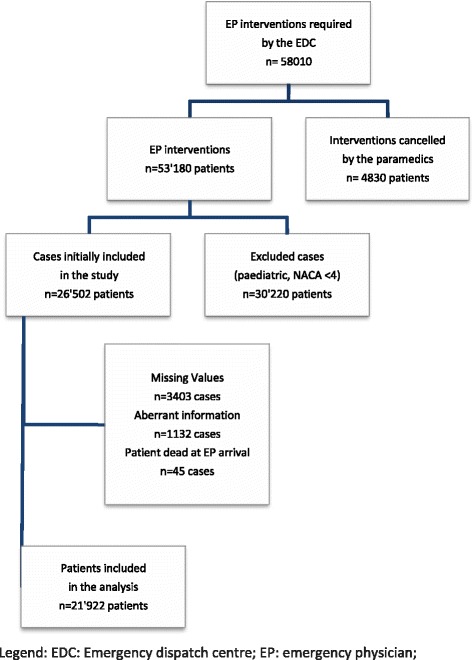
Flow diagram of patients included in the study

We included all missions for adult patients with a National Advisory Committee for Aeronautics (NACA) score of ≥ 4 (Fig. 1), who received care during a EP-staffed EMS intervention [16]. Missions including patients who were considered dead on EP arrival and those cancelled by paramedics were excluded.

### Database, outcomes and exposures

For each EMS mission, a standardized medical report was collected in a central anonymous data registry, as previously described [2]. These data sets included Utstein variables for uniform reporting of cardiac arrest, and they were in accordance with the Utstein recommended data set for major trauma and with the Uniform Pre-Hospital Emergency Medical Services Data Conference guidelines [17–19]. Out-of-hospital degree of case severity was estimated by using the NACA score [16]. The NACA score comprises seven categories, ranging from 0 (no injury or disease) to 7 (lethal injuries or diseases, with or without resuscitation attempts). NACA scores of ≥ 4 imply potential life-threatening conditions [16].

ADMI were defined according to the out-of-hospital procedures and medical treatment performed on site or during transport to the hospital. ADMI included the following: cricothyroidotomy, endotracheal intubation, supraglottic device use, external chest compression (manual or with a mechanical device), defibrillation, electrical cardioversion, external pacing, intraosseous access, haemostatic “tourniquet” and pneumothorax needle decompression. Advanced medical treatments included adenosine, atropine, amiodarone, ephedrine, epinephrine, vasopressin, etomidate, flumazenil, naloxone, ketamine, succinylcholine and/or vecuronium. ADMI was considered as a binary variable, taking a value 1 if the patient received ≥ 1 invasive procedure or emergency treatment. Our outcome was defined as the probability of receiving ADMI.

Manual upper airway opening, bag and mask ventilation, Heimlich manoeuvre, oxygen, salbutamol aerosol, peripheral intravenous access, crystalloid infusion, compression bandage, standard cardiopulmonary monitoring, 12-lead electrocardiogram, and any oral or intravenous medication other than those mentioned earlier were considered standard interventions and thus accounted for non-ADMI.

We first investigated the probability of having any ADMI in the whole group of patients considered in this study. We were then interested in assessing this probability among two subgroups of patients. The first subgroup consisted of those patients who had an indication for intubation and/or invasive upper airway management, defined in our study as a Glasgow Coma Scale (GCS) score of 3/15 or the presence of head trauma with a GCS score of ≤ 8/15. ADMI assessed in that subgroup included endotracheal intubation, failure of endotracheal intubation, cricothyroidotomy, supraglottic devices and medications for rapid sequence induction of anaesthesia: etomidate, ketamine, succinylcholine, vecuronium.

The second subgroup of patients consisted of those who had a cardiac arrest and thus an indication for cardiopulmonary resuscitation. Patients in cardiac arrest were identified by a documented Utstein data set and/or an initial rhythm of ventricular fibrillation or pulseless tachycardia (VF/pVT), asystole or pulseless electrical activity (PEA), and/or an initial diagnosis of cardiac arrest. ADMI assessed in that subgroup included external chest compressions, defibrillation, epinephrine, amiodarone and/or vasopressin.

For all analyses, we considered the following independent variables: gender, diagnostic categories at 48 h (cardiovascular, trauma, pulmonary, neurologic, intoxication, psychiatric, obstetric, miscellaneous), site of EMS mission (patient’s home, public place, nursing home, physician’s office), time of the mission (day: 8:00-17:59, evening: 18:00-23:59, or night: 00:00-07:59) and destination decision (admitted to Lausanne University Hospital Emergency Department wards or shock room, admitted to another regional hospital, transferred to another region, transferred to the helicopter team, death on site, alive not transported). Initial vital parameters comprised heart rate (0–60/min, 61–100/min, >100/min), respiratory rate (0–8/min, 9–20/min, > 20/min), SpO_2_ (< 89 %, ≥ 89 %), systolic blood pressure (0–89 mmHg, 90–150 mmHg, > 150 mmHg), GCS (3–15) and the presence of cardiac arrest (yes/no). Age deciles, i.e., age groups accounting for 10 % of the whole population (18–37, 38–49, 50–58, 59–64, 65–70, 71–75, 76–79, 80–83, 84–88, ≥ 89 years) were considered in the bivariate and multivariate analyses.

For the subgroup analysis of patients in cardiac arrest, additional independent variables were considered: initial rhythm of the cardiac arrest (asystole, ventricular fibrillation or pulseless tachycardia, pulseless electrical activity); presumed origin of cardiac arrest (cardiac, not cardiac); witnessed by bystander, witnessed by the EMS team, or not witnessed; chest compressions by the witness or not; time of arrest to defibrillation < 8 min or > 8 min.

### Statistical analysis

Bivariate analyses were performed to assess the association of age with outcomes. We determined whether the frequency of ADMI was associated with the patient’s age, independently of other factors. ADMI was analysed as a dichotomous variable that took the values of 0 or 1, as described earlier. Missions recorded with at least one missing value in the variables used in the study were excluded. A logistic regression analysis was performed to assess the association of the outcome with age. We performed logistic regression models with a backward selection procedure to select the regressors to be included in the model. More specifically, we started with a saturated model that included all two-way interactions between the variable age (categorical) and each independent variable. A series of Wald tests were performed (using 5 % for type 1 error probability) to select the statistically significant interactions. The variable age was in turn tested. No adjustment for multiple testing was performed, as the study was descriptive [20]. The NACA score was not included in the model because it was assessed after the mission. For subgroup analyses, we could not start with the saturated model (i.e., to test all the interactions at the same time) because of the smaller sample size. Rather, we tested each interaction in turn and retained only the statistically significant interactions. The same modelling procedure was performed for the secondary outcome. Analyses were conducted by using STATA statistical software v.13.1 (STATA Corp., College Station, Texas, USA). The level of significance was a *p* value < 0.01.

## Results

Between 2005 and 2013, a EP-staffed EMS was dispatched by the emergency dispatch centre in 58,010 situations. Of these, 21,922 cases were available for analyses (Fig. 1).

The most frequent diagnoses were cardiovascular (50 % of cases), neurologic (13 %) and pulmonary (12 %) conditions (Table 1). Only 13 % of the patients aged 18–37 years had a cardiovascular diagnosis, whereas it constituted the majority (58 %) of the situations in patients aged 76 years and over. Inversely, the proportion of trauma diagnoses was largest in the younger age groups and smallest in the older age groups. The probability of a mission at home or in nursing homes increased progressively with advancing age, whereas trauma requiring an EP decreased with advancing age.

**Table 1:** Patients and missions characteristics according to age categories, for out-of-hospital missions performed in one Swiss region during the years 2005–2013

Percentiles of age (n)	18–37	38–49	50–58	59–64	65–70	71–75	76–79	80–83	84-88	≥ 89	Total
Total n (%)	2243 (10.2)	2205 (10.1)	2401 (10.9)	2069 (9.4)	2256 (10.3)	2203 (10.1)	2043 (9.3)	2177 (9.9)	2495 (11.4)	1830 (8.4)	21922 (100)
Gender n (%)											
Men	1405 (62.6)	1402 (63.6)	1562 (65.1)	1402 (67.7)	1406 (62.3)	1348 (61.2)	1154 (56.5)	1087 (49.9)	1148 (46.0)	668 (36.5)	12582 (57.4)
NACA Score^a^ n (%)											
NACA 4	1362 (60.7)	1363 (61.8)	1436 (59.8)	1188 (57.4)	1326 (58.8)	1244 (56.5)	1222 (59.8)	1264 (58.0)	1497 (60.0)	1089 (59.5)	12991 (59.3)
NACA 5	671 (29.9)	554 (25.1)	612 (25.5)	572 (27.6)	582 (25.8)	644 (29.2)	536 (26.2)	646 (29.7)	726 (29.1)	581 (31.7)	6124 (27.9)
NACA 6	127 (5.7)	163 (7.4)	154 (6.4)	125 (6.0)	154 (6.8)	121 (5.5)	117 (5.7)	111 (5.1)	103 (4.1)	54 (2.9)	1229 (5.6)
NACA 7	83 (3.7)	125 (5.7)	199 (8.3)	184 (8.9)	194 (8.6)	194 (8.8)	168 (8.2)	156 (7.2)	169 (6.8)	106 (5.8)	1578 (7.2)
Diagnosis n (%)											
Cardiovascular	305 (13.6)	863 (39.1)	1 196 (49.8)	1 096 (52.9)	1 253 (55.5)	1 214 (55.1)	1 178 (57.6)	1 251 (57.5)	1 465 (58.7)	1 063 (58.1)	10 884 (49.6)
Neurologic	268 (11.9)	265 (12.0)	337 (14.0)	264 (12.7)	311 (13.8)	288 (13.1)	265 (12.9)	299 (13.7)	378 (15.2)	278 (15.2)	2 953 (13.5)
Pulmonary	133 (5.9)	119 (5.4)	177 (7.4)	263 (12.7)	297 (13.1)	346 (15.7)	326 (15.9)	336 (15.4)	347 (13.9)	256 (13.9)	2 600 (11.8)
Trauma	725 (32.3)	372 (16.9)	256 (10.7)	157 (7.6)	97 (4.3)	92 (4.2)	62 (3.0)	71 (3.3)	76 (3.0)	64 (3.5)	1 972 (9.0)
Intoxication	483 (21.5)	322 (14.6)	137 (5.7)	44 (2.1)	37 (1.6)	30 (1.3)	19 (0.9)	17 (0.8)	23 (0.9)	21 (1.1)	1 133 (5.2)
Psychiatric	33 (1.5)	12 (0.5)	12 (0.5)	8 (0.4)	3 (0.1)	1 (0.1)	4 (0.2)	5 (0.2)	4 (0.1)	2 (0.1)	84 (0.4)
Obstetric	60 (2.7)	17 (0.8)	3 (0.1)	0 (0.0)	0 (0.0)	0 (0.0)	0 (0.0)	0 (0.0)	0 (0.0)	0 (0.0)	80 (0.4)
Miscellaneous	236 (10.5)	235 (10.7)	283 (11.8)	237 (11.5)	258 (11.4)	232 (10.5)	189 (9.2)	198 (9.1)	202 (8.1)	146 (7.9)	16 (10.1)
Place of intervention n (%)											
Home	814 (36.3)	1099 (49.8)	1402 (58.4)	1304 (63.0)	1565 (69.4)	1572 (71.4)	1472 (72.1)	1538 (70.6)	1736 (69.6)	1207 (65.9)	13709 (62.5)
Nursing Home	10 (0.5)	22 (1.0)	39 (1.6)	42 (2.0)	79 (3.5)	104 (4.7)	116 (5.7)	192 (8.8)	307 (12.3)	372 (20.3)	1283 (5.8)
Public Place	1075 (47.9)	737 (33.4)	599 (24.9)	386 (18.7)	298 (13.2)	237 (10.7)	179 (8.8)	167 (7.7)	151 (6.1)	90 (4.9)	3919 (17.9)
Physician office	51 (2.3)	112 (5.1)	155 (6.4)	122 (5.9)	114 (5.0)	92 (4.2)	86 (4.2)	58 (2.7)	67 (2.7)	32 (1.7)	889 (4.0)
Other	293 (13.1)	235 (10.7)	206 (8.6)	215 (10.4)	200 (8.9)	198 (8.9)	190 (9.3)	222 (10.2)	234 (9.4)	129 (7.1)	2122 (9.7)
Destination n (%)											
Lausanne University Hospital, ED^b^ wards	500 (22.3)	427 (19.4)	444 (18.5)	371 (17.9)	375 (16.6)	394 (17.9)	397 (19.4)	403 (18.5)	500 (20.0)	496 (27.1)	4 307 (19.6)
Lausanne University Hospital, Shock room	692 (30.8)	685 (31.1)	739 (30.8)	635 (30.7)	659 (29.2)	632 (28.7)	522 (25.6)	510 (23.4)	595 (23.8)	317 (17.3)	5 986 (27.3)
Other regional hospital in the region	885 (39.5)	897 (40.7)	940 (39.2)	809 (39.1)	948 (42.0)	922 (41.8)	909 (44.5)	1 059 (48.6)	1 198 (48.0)	868 (47.4)	9 435 (43.0)
Transferred in another region	42 (1.9)	45 (2.0)	49 (2.0)	45 (2.2)	43 (1.9)	35 (1.6)	31 (1.5)	21 (0.9)	16 (0.6)	13 (0.7)	340 (1.5)
Transferred to the helicopter team	28 (1.3)	12 (0.5)	9 (0.4)	6 (0.3)	7 (0.3)	2 (0.1)	1 (0.0)	2 (0.09)	2 (0.1)	1 (0.1)	70 (0.3)
Death on site	79 (3.5)	119 (5.4)	196 (8.1)	181 (8.8)	192 (8.5)	192 (8.7)	168 (8.2)	156 (7.2)	163 (6.5)	105 (5.7)	1 551 (7.1)
Not transported, alive	8 (0.4)	17 (0.8)	16 (0.7)	12 (0.6)	19 (0.8)	14 (0.6)	8 (0.4)	20 (0.9)	11 (0.4)	20 (1.1)	145 (0.6)
Time of intervention n (%)											
Day: 8:00-17:59	783 (34.9)	891 (40.4)	1 048 (43.6)	903 (43.6)	974 (43.2)	1 001 (45.4)	942 (46.1)	1 038 (47.7)	1 185 (47.5)	908 (49.62)	9 673 (44.1)
Evening: 18:00-23:59	888 (39.6)	903 (40.9)	877 (36.5)	752 (36.3)	797 (35.3)	714 (32.4)	648 (31.7)	650 (29.9)	759 (30.4)	589 (32.19)	7 577 (34.6)
Night: 00:00-07:59	572 (25.5)	411 (18.6)	476 (19.8)	414 (20.0)	485 (21.5)	488 (22.2)	453 (22.2)	489 (22.5)	551 (22.1)	333 (18.2)	4 672 (21.3)

NACA score^a^ [16]^:^ NACA 0: No injury or disease, NACA 1: Injuries/diseases without any need for acute physicians care, NACA 2: Injuries/diseases requiring examination and therapy by a physician, but hospital admission is not indicated, NACA 3: Injuries/diseases without acute threat to life but requiring hospital admission, NACA 4: Injuries/diseases that can possibly lead to deterioration of vital signs, NACA 5: Injuries/diseases with acute threat to life, NACA 6: Injuries/diseases transported after successful resuscitation of vital signs, NACA 7: Lethal injuries or diseases (with or without resuscitation attempts)

ED^b^: emergency department

The probability of having an ADMI decreased with age (Table 2), being highest from 18 to 58 years, lower between 59 and 88 years (odds ratio [OR] varying between 0.80 and 0.85) and significantly lower for those aged 89 years and over (OR = 0.66; 95 % CI: 0.53–0.82).

**Table 2:** Adjusted odds ratios^b^ and 95 % confidence intervals for having an advanced medical intervention according to age in out-of-hospital EP missions performed in one Swiss region during the years 2005–2013

		Odds ratios (OR)	95 % Confidence interval (CI)	*P*-value
Age^a^	18–37	(1.00)		
	38–49	0.97	0.81–1.15	0.71
	50–58	1.01	0.84–1.21	0.94
	59–64	0.85	0.70–1.03	0.10
	65–70	0.87	0.71–1.06	0.17
	71–75	0.86	0.71–1.05	0.15
	76–79	0.79	0.64–0.97	0.03
	80–83	0.88	0.72–1.07	0.20
	84–88	0.80	0.65–0.97	0.02
	≥89	0.66	0.53–0.82	<0.001

^a^Deciles

^b^Adjusted for gender, cardiac arrest, categories of diagnosis, Glasgow Coma Scale, heart rate, respiratory rate, systolic blood pressure

EP: emergency physician

Level of significance: *p* < 0.01

**Fig. 2:**
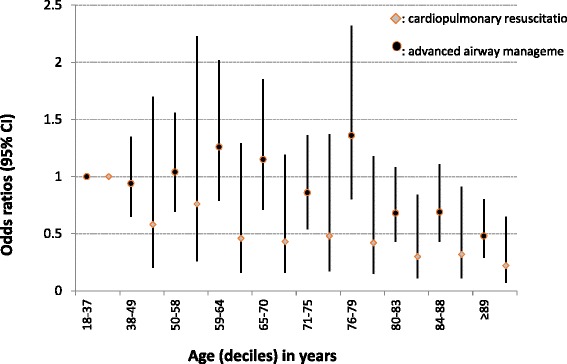
Adjusted odds ratio and 95 % confidence intervals for cardiopulmonary resuscitation and advanced airway management, according to age, in out-of-hospital EP missions performed in one Swiss region during the years 2005–2013

Among the subgroup of patients in cardiac arrest (*n* = 1,680), the probability of cardiopulmonary resuscitation attempts decreased progressively with age. For the three oldest age deciles (80–83, 84–88 and ≥ 89 years), the probability of having cardiopulmonary resuscitation was significantly lower (Fig. 2). Among patients who were candidates for advanced airway control (*n* = 2,997), the probability of being intubated did not vary significantly by age decile except for patients aged 89 years and over (OR = 0.5; 95 % CI: 0.3–0.8) (Fig. 2).

## Discussion

In this study, we showed that the rate of ADMI performed during EP-staffed EMS missions varied significantly with age. ADMI probability, whatever the procedure or treatment, was the highest in patients aged 58 years and younger, slightly lower between ages 59 and 88, and significantly lower for patients aged 89 and over. In patients with a cardiac arrest, a decrease in resuscitation procedures was also observed in patients aged 80 and over. Half of the situations requiring an EP-staffed EMS mission were related to cardiovascular problems. For patients ≥ 80 years, cardiovascular diagnoses constituted the majority of the missions, whereas they involved only 13 % of the diagnoses observed in the group who were 18–37 years old.

Variations observed across age groups suggest the hypothesis that age might play a role in medical decision-making to initiate ADMI or not in out-of-hospital emergency situations, at least in the surveyed region. According to previous studies, decreases in ADMI, particularly in patients aged 80 and over, may indicate that these interventions could be considered as increasingly futile with advancing age [2]. Previous studies have illustrated similar biphasic response curves, with a progressive increase in emergency procedures such as airway management to a maximum between 50 and 70 years, followed by a decrease from 70 or 80 years [7]. This inflection point may illustrate the subjective and individual feeling of EMS providers, including EP, that patients are becoming “too old to benefit” from ADMI. This inflection point may vary across communities, depending on the “age” of the population and the experiences and skills of EMS providers. Nevertheless, we cannot exclude the possibility that in our study, age might have been acting only as a “surrogate marker” of frailty and severe comorbidities.

In accordance with previous studies, [2, 21] cardiovascular problems were the most frequent diagnoses observed in out-of-hospital EP missions, followed by respiratory and neurological emergencies. Previous studies have demonstrated that older patients with acute ischaemic cardiac diseases were less likely to benefit from cardiac catheterization or other invasive procedures [22–24]. In our study, in patients who sustained a cardiac arrest, the probability of cardiopulmonary resuscitation progressively decreased with age, particularly after 80 years. Again, our data do not allow for a direct analysis of the comorbidities of the patients and age may thus be an indirect indicator of illness, dependency or frailty, not identified in our study, but possibly evaluated by the out-of-hospital emergency team on site. Some studies have tended, nevertheless, to indicate that in time-sensitive emergencies such as cardiac arrest situations, the limited amount of time available for evaluation more frequently led to decisions being made that were based on simple characteristics such as age [25].

Wong et al. [26] observed the way in which the incidence of out-of-hospital cardiac arrest and survival evolved between 2002 and 2010. Among their main observations, they noted an improvement in survival in younger patients and a decrease in survival in older patients. These authors suggested that this finding may be accounted for by differences in terms of treatment aggressiveness for different age groups, as well as greater physiological reserve to tolerate periods of hypoperfusion among younger patients. The decrease in the rate of shockable rhythm with age at initial presentation should also be taken into account [27].

### Limitations and strengths

This study is subject to several limitations. Some important variables related to past medical illnesses, comorbidities and the clinical state of the patient were not collected and could therefore not be considered for analysis. Moreover, detailed information about the will of the patient (or relatives or proxies) about ADMI or the presence of advance directives were not documented, which potentially restricted the interpretation of the results. In terms of generalizability, the results may apply to other Western European countries with similar EP-staffed EMS, but cannot be generalized to other countries because of the differences in EMS models. Strengths were the large sample size and the exhaustive data on EMS use and on-site interventions.

## Conclusion

The medical decision to initiate ADMI in out-of-hospital emergency situations varied with age and decreased in older patients, particularly in those ≥ 89 years. This finding warrants further analysis of the decision-making processes in the context of out-of-hospital emergencies for older patients. Further quantitative and qualitative studies are needed to investigate whether this observation corresponds to underuse of ADMI in very old patients.

## Abbreviations

ADMI: Advanced medical intervention(s)
CI: Confidence interval
EMS: Emergency medical service(s)
EP: Emergency physician(s)
GCS: Glasgow coma scale
NACA score: National Advisory Committee for Aeronautics score
OR: Odds ratio
